# Programmable Photonic Logic Array Based on Micro-Ring Resonators and All-Optical Modulation

**DOI:** 10.3390/mi16020238

**Published:** 2025-02-19

**Authors:** Jia Liu, Shenghang Zhou, Xiubao Sui

**Affiliations:** 1 Jiangsu Key Laboratory of Spectral Imaging and Intelligent Sense, Nanjing University of Science and Technology, Nanjing 210094, China; jialiu@njust.edu.cn (J.L.); zsh_njust@163.com (S.Z.); 2 School of Electronic and Optical Engineering, Nanjing University of Science and Technology, Nanjing 210094, China

**Keywords:** nonlinear micro-ring resonator, optical standard logic unit, programmable photonic logic array, optical logic computing

## Abstract

All-optical computing is an emerging information processing technology. As a cutting-edge technology in the field of photonics, it effectively leverages the unique advantages of photons to achieve rapid computation. However, the lack of a fully functional and programmable design has slowed the progress of this type of optical computing system, especially in optical logic computing. In this paper, we design and propose a programmable photonic logic array based on all-optical computing methods. By efficiently combining on-chip photonic devices such as micro-ring resonators, we have realized a complete set of reconfigurable all-optical logic computation functions, including basic logic such as IS&NOT, AND, and OR, as well as combined logic, such as XOR and XNOR. To the best of our knowledge, the proposed architecture not only introduces three structurally similar standard logic units but also allows for their multiple-level cascading to form a large-scale photonic logic array, enabling multifunctional logic computation. Furthermore, using two independent wavelengths to represent the high and low levels of logic can effectively reduce cross-talk and overlap between signals, decreasing the dependence on the strength of the optical signal and the decision threshold. Simulation results by Photonic Integrated Circuit Simulator (INTERCONNECT) demonstrate the effectiveness and feasibility of the proposed programmable photonic logic array.

## 1. Introduction

Thanks to advancements in manufacturing processes, photonic integrated chips are rapidly developing, offering advantages such as low latency, high transmission, strong anti-interference capabilities, and ultra-low power consumption, effectively overcoming electronic bottlenecks in power consumption, interconnectivity, and latency [1,2,3]. These advancements are set to revolutionize new media and information processing methods, such as optical neural networks (ONNs) [4,5,6,7], optical filtering, and routing. These developments are extensively applied in fields like computing, information, and image processing, as well as communication. In the era of big data, optical neural networks have been a mainstream direction for information processing. As a large-scale photonic integrated circuit, they are used to perform dense matrix multiplication to accomplish tasks such as image classification, object tracking, and natural language recognition. However, these optical neural network chips are usually tailor-made for specific neural network algorithms and operate in an analog signal manner, lacking in reconfigurability, functionality, and complexity control, and they lack universal adaptability for complex computational tasks. Therefore, in scenarios requiring high precision and complex data processing, optical digital computing is preferred and widely used for its accuracy, universality, and programmable flexibility. This requires a large number of optical logic units and their high integration into large-scale photonic logic arrays.

In recent years, numerous schemes for realizing optical logic operations have emerged, which can be broadly categorized into two types, namely, optical guided logic and all-optical logic. The optical guided logic utilizes an electrical signal as the logic operand loaded onto MRR to control the resonance state, which in turn, directs the flow of the input optical signals, and ultimately, the optical power output of the particular port is considered as the result of the optical logic, indicating the logic function, thereby realizing a specific logic function. In 2010 and 2011, Tian et al. proposed various optical structures based on cascading multiple micro-ring resonators to realize optical computing functions such as XOR/XNOR, AND/NAND, and OR/NOR by [8,9]. From 2011 to 2019, they successively developed structures for half-adder/subtractor [10,11,12], encoders [13,14], decoders [15], comparators [16,17,18], parity checkers [19], Feynman gates [20], among others. In 2024, Chen et al. introduced an ultra-compact optical full adder, significantly reducing the footprint by using only four MRRs [21]. In the same year, Xia et al. proposed an ultra-compact and multifunctional on-chip optical computing system, achieving optical computing functions including optical decoder, half adder, and multiplier [22]. Different from optical guided logic, which uses electrical signals for controlling, the all-optical logic uses light itself as the logic variable to change the resonance state of MRR based on optical nonlinear effects, and then controls the propagation of the input optical signal in the optical path, and ultimately realizes the all-optical logic computing. In 2007, Xu et al. realized AND and NAND logic functions using a single micro-ring, based on resonance wavelength drift due to four-wave mixing and two-photon absorption effects [23]. Between 2017 and 2022, Dong and colleagues utilized four-wave mixing (FWM) [24,25] and cross-gain modulation (XGM) [26] in semiconductor optical amplifiers, as well as FWM in higher-order nonlinear optical fibers [27], to implement basic logical functions, including AND, OR, NOT, XOR, XNOR, NAND, and NOR, which they named as standard logic units. In addition, four-wave mixing in nonlinearly enhanced silicon waveguides [28], sum-frequency generation (SFG), difference-frequency generation (DFG), and second-harmonic generation (SHG) [29,30] in periodically poled lithium niobate (PPLN), and transient cross-phase modulation (T-XPM) [31] have also been explored for the realization of all-optical logic.

With the development of optical computing technology and photonic integration technology, complex computations can be achieved by combining the design of simple computational functions with device integration to form photonic circuits. Programmable logic arrays will have even more significant advantages, as they integrate multiple optical logic units that can switch configuration states based on task requirements. This provides a high degree of flexibility and reconfigurability, enabling the execution of various complex computational tasks. Based on the optical guided logic method, in 2017, Singh et al. designed a reconfigurable cellular optically guided logic structure based on optical switches for micro-ring resonators that requires photoelectric conversion for the receiver [32]; in 2016, Tian Y proposed a reconfigurable guided logic computational structure [33] that utilizes a single waveguide and an array of micro-rings based on the principle of incoherent superposition to achieve any logic operation of the optical switch array which not only performs arbitrary logic operations based on logic expressions but also integrates multiplexing/demultiplexing functions, thereby reducing the complexity of logic circuits. For the all-optical logic method, from 2004 to 2022, Huazhong University of Science and Technology (HUST) has conducted extensive research on the design of semiconductor optical amplifier-based logic circuits on silicon-on-insulator (SOI) nanowires, achieving the full-flow development of logic circuits from basic logic gates to advanced logic circuits. Building on this foundation, HUST has explored the scheme design of multi-input configurable logic units-programmable logic arrays (CLU-PLAs) implemented via various nonlinear optical techniques and corresponding capacity expansion research. The performance, limitations, and integration of the extended CLU-PLA schemes have also been thoroughly discussed [2,3,25,34,35,36,37].

Optical guided logic, which uses electrical signals as operational logic numbers, is easy to modulate through external electrical control signals, offering good controllability and tunability. However, due to this reliance on electrical signals, it will have limitations in speed, bandwidth, and performance. Additionally, it requires optoelectronic conversion, which increases system complexity and latency. On the other hand, all-optical logic operates directly on optical signals without the need for optoelectronic conversion, thus avoiding the shortcomings of optical guided logic. It achieves high-speed, low-energy, high-bandwidth, and low-latency computational performance, and can even perform parallel computing through multipath transmission. However, current implementations of all-optical logic units and arrays are mostly based on four-wave mixing in semiconductor optical amplifiers (SOAs) or higher-order nonlinear fibers, as well as cross-phase/gain modulation. Although these approaches have the inherent advantages of optical computing, they suffer from large footprints, high pump energy, low nonlinear efficiency, and complex design requiring precise control. To address these issues, we have chosen micro-ring resonators as the core, utilizing the two-photon absorption effect to implement all-optical logic computation. This approach not only retains the inherent characteristics of high-speed response, low energy consumption, and parallel processing but also offers additional advantages such as small size, high integration, strong reconfigurability, low pump power, and high nonlinear efficiency, which are brought about by the structure, tuning characteristics, and two-photon absorption of the micro-ring resonators.

In this paper, we design a set of wavelength-based Optical Standard Logic Units (OSLUs) leveraging optical nonlinear effects and micro-ring resonators (MRRs), and construct a two-input Programmable Photonic Logic Array (PPLA) based on the sum-of-products formed and the proposed wavelength-type OSLUs, combined with other on-chip devices like Mach–Zehnder interferometers (MZIs), to fully realize functions and programmability. By cascading 2–3 MRRs, the OSLUs are constructed and capable of performing fundamental optical computing functions, such as IS, NOT, AND, and OR. Through series and parallel connections of the OSLUs, a PPLA is established, capable of realizing arbitrary logic functions that are reconfigurable and programmable. Compared with the proposed schemes of PPLA, our scheme has several improvements and advantages. First, the MRR is used as the core photonic device in our structure, which has the advantage of a small size and can be highly integrated; second, the proposed OSLUs have similar structure, requiring only the configuration of the initial state of the MRRs, making it easy to operate; third, the realized PPLA is a multilevel cascading of OSLUs, and its programmability and functional reconstruction can be achieved through the MZIs without frequently changing the resonance state of the micro-ring; fourth, the PPLA can achieve precise digital operations, avoiding the cumulative errors common in optical analog operations, and facilitates cascading.

This paper is organized as follows: Section 1 reviews the significance and historical development of optical logic. Section 2 provides a detailed description of the design concepts and working principles of optical standard logic units and programmable photonic logic array. Section 3 outlines the simulation process and presents comprehensive simulation designs and results. Finally, Section 4 summarizes the findings of this paper and discusses potential directions for future research.

## 2. Design Principle

### 2.1. Nonlinear MRR

The basic on-chip device of the proposed optical computing systems is a nonlinear MRR. The working principle of the proposed nonlinear MRR can be summarized as follows: The nonlinear waveguide is embedded into the MRR; if the nonlinear MRR is pumped by strong light, the refractive index of the nonlinear waveguide will change, the MRRs’ resonance state will be switched, and the resonance peak will shift to another wavelength.

**Figure 1:**
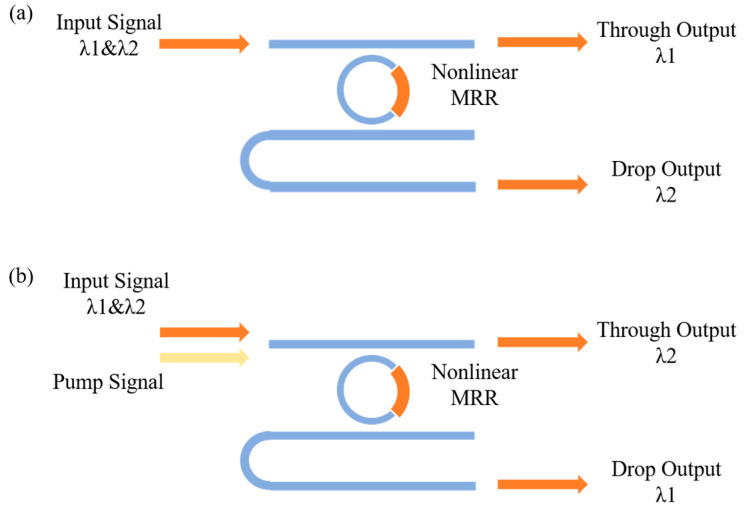
Diagram of the proposed nonlinear MRR. (**a**) Without pump light. (**b**) With pump light.

Specific to this work, as shown in Figure 1, the input optical signal with a wavelength $\lambda_{1}$ and $\lambda_{2}$, stands for the 0 and 1 states of the digital signal, corresponding to a low level and high level, respectively. When there is no pump light, the nonlinear MRR remains in its initial state, the THROUGH port outputs the light with the wavelength $\lambda_{1}$, and the DROP port outputs the light with the wavelength $\lambda_{2}$, as shown in Figure 1a. On the contrary, when the pump light enters into the micro-ring, it produces a modulation of the MRR, changing the resonance state. Under the appropriately designed parameters, the resonance state shifts to $\lambda_{1}$. Note that, as shown in Figure 1b, the THROUGH port outputs light with a wavelength $\lambda_{2}$, and the DROP port outputs light with a wavelength $\lambda_{2}$. Therefore, by utilizing the modulation effect of nonlinear MRR and optical pumping, it is possible to establish a conversion relationship between the input and output signal wavelengths, thereby representing the logical transformation between digital 0 and 1.

### 2.2. Optical Standard Logic Unit (OSLU)

Based on the designed basic structure as shown in Figure 1, the OSLUs are demonstrated. The proposed OSLUs include two types, aiming to implement the optical computing functions of IS&NOT and AND&OR, respectively.

#### 2.2.1. The OSLU for IS&NOT

**Figure 2:**
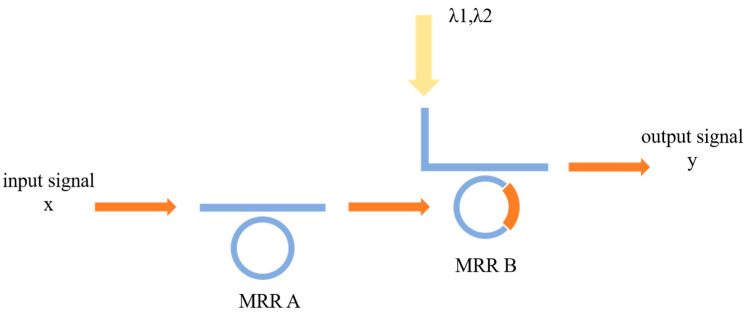
The proposed OSLU for IS&NOT function.

The function IS&NOT can be mathematical expressed as follows:

(1) y=x

(2) $$y=\bar{x}$$

where x is the input signal, $\bar{x}$ denotes the opposite operation, and y is the output signal. The proposed OLSU for IS&NOT is illustrated in Figure 2.

For the IS function, the initial resonant wavelength of MRR A is set as $\lambda_{1}$, and resonant wavelength of MRR B is $\lambda_{2}$ in the absence of pump light, and that is $\lambda_{1}$ in the presence of pump light, respectively. In this configuration, the specific implementation is as follows. Case 1: When the wavelength of the input optical signal x is $\lambda_{1}$, representing value 0, the input signal x is filtered by MRR A, preventing any light from reaching the nonlinear MRR B. Therefore, the wavelength $\lambda_{1}$ is selected and detected at the wavelength of the input signal is $\lambda_{2}$, representing the value 1, it passes through MRR A and illuminates the nonlinear MRR B. MRR B is optically pumped and selects wavelength $\lambda_{2}$, indicating the output y=1. In other words, the result at the THROUGH port represents the function y=x, meaning the computational function IS is realized by the proposed OSLU.

For the NOT function, the initial resonant wavelength of MRR A is changed to $\lambda_{2}$, while MRR B remains unchanged. Similar to the implementation principle of the IS function, the results are the opposite. Case 1: When the wavelength of the input optical signal x is $\lambda_{1}$, representing value 0, the input signal x passes through MRR A and illuminates the nonlinear MRR B. MRR B is optically pumped and selects wavelength $\lambda_{2}$ at the THROUGH port, indicating the output y=1. Case 2: When the wavelength of the input signal is $\lambda_{2}$, representing the value 1, the input signal x is filtered by MRR A, preventing any light from reaching the nonlinear MRR B. MRR B selects the wavelength at the THROUGH port, indicating the output y=0. The result at the THROUGH port represents the function $y=\bar{x}$, meaning the computational function NOT is realized by the OSLU.

#### 2.2.2. The Function of AND

**Figure 3:**
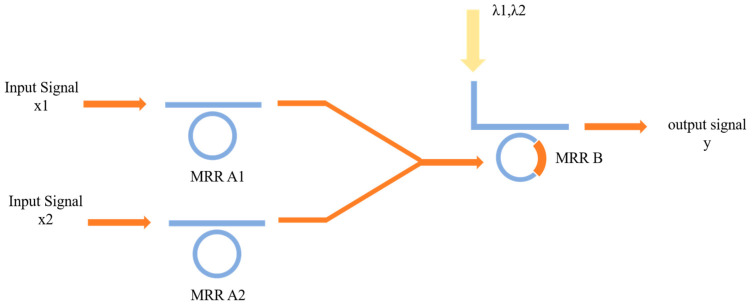
The proposed OSLU for AND function.

The AND function can be expressed as follows:

(3) $$y=x_{1}\cdot x_{2}$$

where $x_{1}$ and $x_{2}$ are the input signals, · and ¯ denote the AND operation and NOT operation, respectively, and y is the output signal representing the result of the operation. The operation of the optical structure is depicted (illustrated) in Figure 3.

For the AND function, the initial resonant wavelength of MRR A1 and MRR B are set to $\lambda_{1}$, and the resonant wavelength of MRR B is $\lambda_{2}$ in the absence of pump light, and that is $\lambda_{1}$ in the presence of pump light, respectively. In this configuration, the specific implementation is as follows. Case 1: when the wavelengths of the input optical signals $x_{1}$ and $x_{2}$ are both $\lambda_{1}$, representing value 0 and 0, respectively, both input signals are filtered by MRR A1 and MRR A2, preventing any light from reaching the nonlinear MRR B. Therefore, the wavelength $\lambda_{1}$ is selected and detected at the THROUGH port of MRR B, standing for the output y=0. Case 2: When the wavelengths of the input signals $x_{1}$ and $x_{2}$ are both $\lambda_{2}$, representing the value 1 and 1, both input signals pass through MRR A1 and MRR A2, and illuminate the nonlinear MRR B. MRR B is optically pumped and selects wavelength $\lambda_{2}$, indicating the output y=1. Case 3: When the wavelengths of the input signals $x_{1}$ and $x_{2}$ are $\lambda_{1}$ and $\lambda_{2}$ respectively, representing the value 0 and 1, at the moment, one input will pass through MRR A1, while the other input will be filtered by MRR A2. However, there is not enough light energy illuminating the nonlinear MRR B to incite nonlinearity. Hence, MRR B is not pumped, and its resonant frequency will remain unchanged at wavelength $\lambda_{2}$. This results in the filtering of the input signal of MRR B at wavelength $\lambda_{2}$, and the optical signal at THROUGH port will be at wavelength $\lambda_{1}$, representing the output y=0. Case 4: When the wavelengths of input signals $x_{1}$ and $x_{2}$ are $\lambda_{2}$ and $\lambda_{1}$, standing for values 1 and 0, respectively. This case operates under the same principle as Case 3, producing identical results, thus it will not be repeated here. In summary, the result at the THROUGH port represents the function $y=x_{1}\cdot x_{2}$, meaning the computational function AND is realized by the OSLU.

#### 2.2.3. The Function of OR

**Figure 4:**
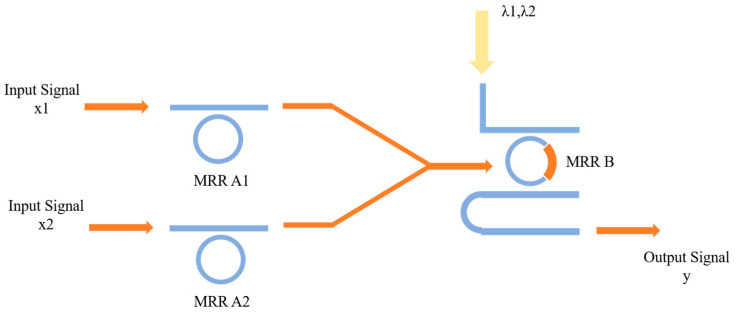
The proposed OSLU for OR function.

The OR function can be expressed as follows:

(4) $$y=x_{1}+x_{2}$$

where $x_{1}$ and $x_{2}$ are the input signals, + and ¯ denote the OR operation and NOT operation, respectively, and y is the output signal representing the result of the operation. The operation of the optical structure is depicted (illustrated) in Figure 4.

For the OR function, the initial resonant wavelength of MRR A1 and MRR B are set as $\lambda_{2}$, and the resonant wavelength of MRR B is $\lambda_{2}$ in the absence of pump light, and that is $\lambda_{1}$ in the absence of pump light, respectively. In this configuration, the specific implementation is as follows. Case 1: When the wavelengths of the input signals $x_{1}$ and $x_{2}$ are both $\lambda_{1}$, representing the value 0 and 0, both input signals pass through MRR A1 and MRR A2, and illuminate the nonlinear MRR B. MRR B is optically pumped and selects wavelength $\lambda_{1}$ at the DROP port of MRR B, indicating the output y=0. Case 2: When the wavelengths of the input optical signals $x_{1}$ and $x_{2}$ are both $\lambda_{2}$, representing values 1 and 1, respectively. Both input signals are filtered by MRR A1 and MRR A2, preventing any light from reaching the nonlinear MRR B. Therefore, the wavelength $\lambda_{2}$ is selected and detected at the DROP port of MRR B, standing for the output y=1. Case 3: When the wavelengths of the input signals $x_{1}$ and $x_{2}$ are $\lambda_{1}$ and $\lambda_{2}$ respectively, representing the value 0 and 1, at the moment, one input will pass through MRR A1, while the other input will be filtered by MRR A2. However, there is not enough light energy illuminating the nonlinear MRR B to incite nonlinearity. Hence, MRR B is not pumped, and its resonant frequency will remain unchanged at wavelength $\lambda_{2}$. This results in the filtering of the input signal of MRR B at wavelength $\lambda_{2}$, and the optical signal at DROP port will be at wavelength $\lambda_{2}$, representing the output y=1. Case 4: When the wavelengths of input signals $x_{1}$ and $x_{2}$ are $\lambda_{2}$ and $\lambda_{1}$, standing for values 1 and 0, respectively. This case operates under the same principle as Case 3, producing identical results, thus it will not be repeated here. In summary, the result at the DROP port represents the function $y=x_{1}+x_{2}$, meaning the computational function OR is realized by the OSLU.

### 2.3. Programmable Photonic Logic Array (PPLA)

The all-optical combinatorial logic is structured as a sum-of-products type, organized into three levels. The first level is the input optical circuit, which employs the IS&NOT function to provide two complementary inputs for the entire photonic circuit. The second level is the minimum term array, which uses the AND function to generate all the minimum terms for the inputs. The third and final level is the coupling array, which utilizes the OR function to select and couple all the minimum terms, thereby realizing any combinational logic output.

**Figure 5:**
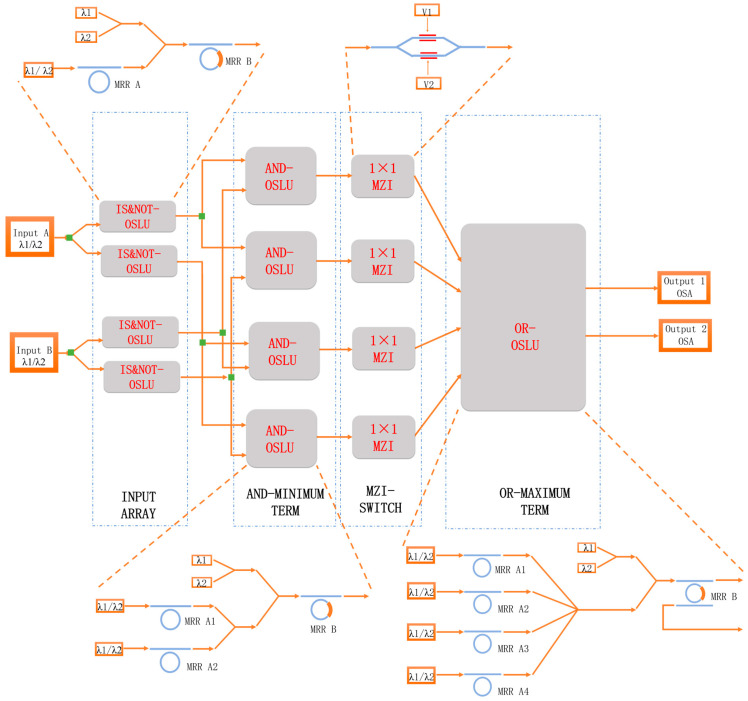
Structure of programmable photonic logic array for implementing arbitrary logic functions.

The proposed PPLA is organized based on the sum-of-products type, utilizing minimum term units. Building on the three optical standard logic units designed in Section 2.2, IS&NOT, AND, and OR, the photonic logic array structure is constructed through multilevel linkage to realize arbitrary combinations of logic functions. The design structure is illustrated in Figure 5.

For the first level, the OSLU depicted in Figure 1 implements the IS&NOT function to perform logic holding and logic negation operations, which provides the necessary absolute and complementary data pattern for the entire photonic array. For the second level, the OSLU shown in Figure 2 implements the AND function, performing AND logic operations for the input signals generated by the first level, producing the minimum terms. For the third level, the OSLU illustrated in Figure 3 implements the OR function, performing OR logic operations on all the minimum terms produced by the second level. In addition, an additional column switch array composed of 1 × 1 MZI is inserted between the second and third levels to selectively choose the minimum terms generated by the AND operations in the second level, allowing for any combination in the OR operations of the third level. The presence of the column switch array makes the PPLA functionally reconfigurable.

Based on the aforementioned operations, we have designed a two-input single-output PPLA. First, two light sources are used as the two input logic variables, with adjustable wavelengths. Second, after the first level of OSLUs implementing the IS&NOT function, complementary logic data streams are generated. Since each input has two logic signals, this level employs four IS&NOT-OSLUs, producing a total of four logical input signals. Then, these four logical signals are fed into the second level of OSLUs implementing the AND function. The two inputs and four logic signals will produce four AND logic combinations, hence this level also uses four AND-OSLUs. Following that, the four AND logic signals enter the column switch array, providing different input combinations for the third-level circuit. Finally, they enter the third level of OSLUs implementing the OR function, generating the final logic output signal. This level only uses one OR-OSLU with four inputs, which can still function properly when the number of inputs is less than four.

## 3. Simulation Results

In order to verify the feasibility and functionality of the designed OSLU and PPLA, this study undertook the construction of models and the execution of simulation tests on the proposed devices and systems. Two resonant wavelengths for the designed MRR are set to $\lambda_{1}$ = 1543.29 nm and $\lambda_{2}$ = 1547.80 nm, respectively, which can be tuned by thermal tuning. In the structural design, the MRR with a radius of 9.775 μm is adopted, with a resonant wavelength of 1543.29 nm. By applying an appropriate voltage and undergoing thermo-optic modulation, the resonant wavelength is tuned to 1547.80 nm. For the nonlinear MRR, a nonlinear region is added and designed to be a quarter of the cavity length, approximately 15.3545 μm. When no light is incident on this region, its resonant state and behavior are identical to those of the linear MRR. When sufficient optical energy is applied, a carrier dispersion effect will occur in the waveguide, changing the waveguide refractive index and thus altering the resonant state of the MRR. With the appropriate parameters given, the resonant wavelength of the nonlinear MRR is 1547.80 nm, and after thermo-optic modulation, the resonant wavelength is changed back to 1543.29 nm. In the design, the two resonant wavelengths are separated by half an FSR distance, ensuring that whether in the initial resonant state under thermal modulation or the tuned resonant state under optical pumping, both are maintained below these two wavelengths.

### 3.1. Micro-Ring Resonator (MRR)

#### 3.1.1. Linear MRR

**Figure 6:**
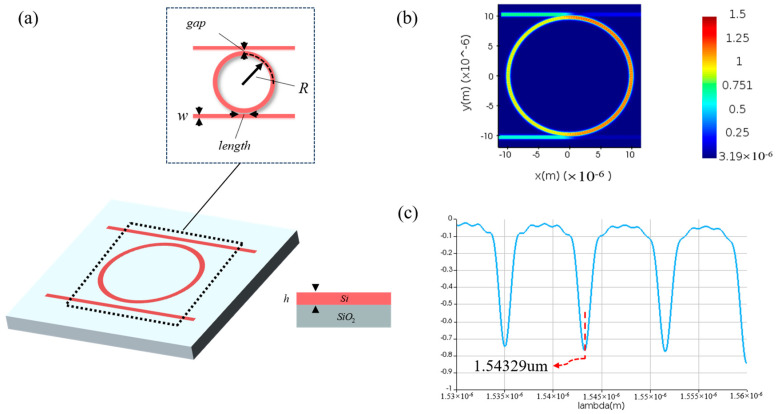
Temperature distribution of MRR. (**a**) Structure for MRR. (**b**) Optical field transmission for MRR. (**c**) Transmission for MRR.

The bending radius, coupling spacing, and coupling length of MRR seriously affect its key characteristics such as resonant wavelength, free spectral range, and Q value. In order to achieve resonance at specific wavelengths, we performed optical simulations of MRR based on the finite-difference time-domain method. Through parameter sweeping, we optimized the MRR design with a bending radius of 9.775 µm, a coupling length of 0.3 nm, and a coupling spacing of 0.1 nm. The resulting resonant wavelength is 1543.29 nm. The structure and optical field transmission of the MRR are illustrated in Figure 6.

**Figure 7:**
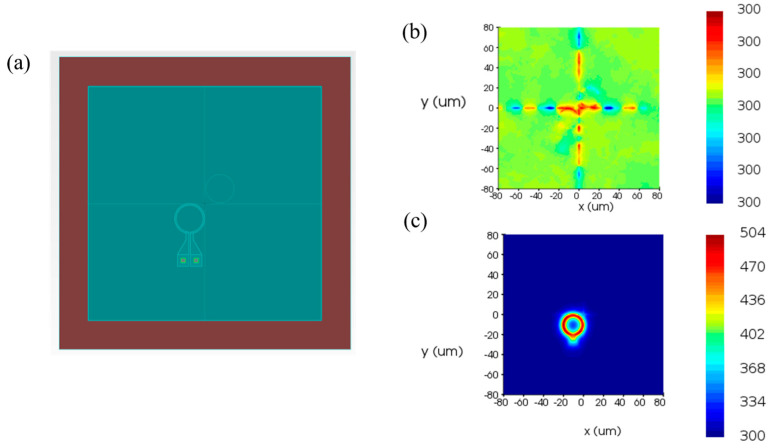
Temperature distribution of MRR. (**a**) Model for thermal simulation. (**b**) Voltage is 0 V. (**c**) Voltage is 2.667 V.

**Figure 8:**
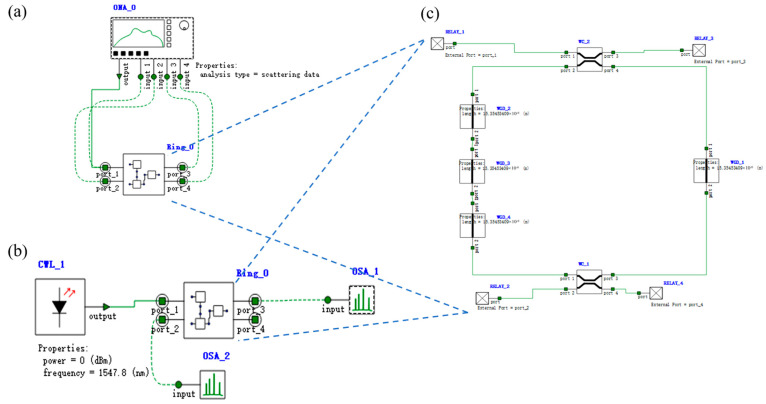
The model for linear MRR. (**a**) Frequency-domain simulation. (**b**) Time-domain simulation. (**c**) Structure of linear MRR. OSA: Optical spectral analyzer. CWL: Continuous wave laser. Ring: Micro-ring resonator. WGD: Straight waveguide. WC: Waveguide directional coupler.

**Figure 9:**
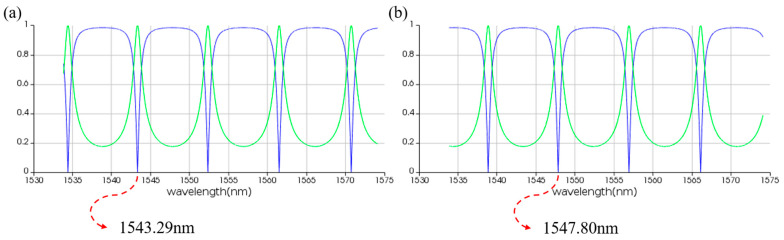
The spectral transmission results for linear MRR in the presence of pumping. (**a**) Without thermal tuning. (**b**) With thermal tuning.

**Figure 10:**
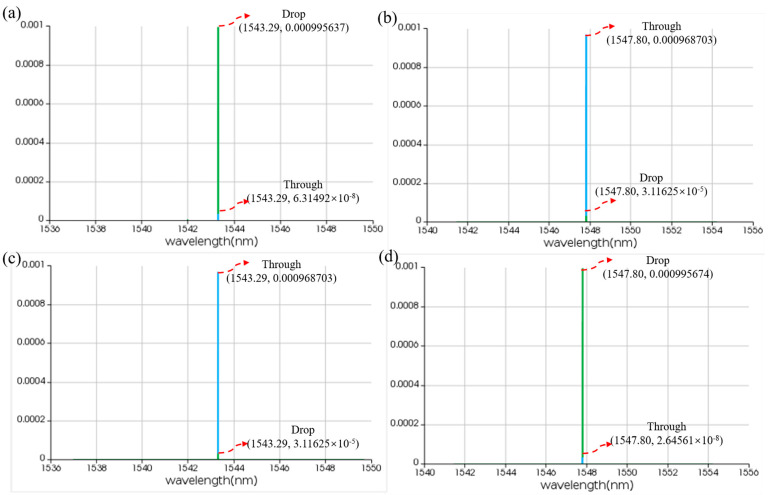
Time-domain simulation results for linear MRR in the presence of pumping. (**a**,**b**) THROUGH and DROP port without thermal tuning. (**c**,**d**) THROUGH and DROP port with thermal tuning. The horizontal axis represents the wavelength unit in nm, and the vertical axis represents the output power unit in W.

In order to achieve an adjustable resonant wavelength, the MRR must be equipped with a modulation function. Here, we choose thermo-optic modulation, which involves applying a voltage to a metal heater to change the temperature of the waveguide, thereby altering its refractive index and achieving MRR resonant wavelength shift. To this end, we performed thermal simulations on the MRR using the finite element method, increasing the modulation voltage from 0 V to 3 V in steps of 0.001 V. Figure 7 illustrates the temperature distribution of the MRR at different voltages. In order to observe the modulation of voltage on MRR, we employed the finite difference eigenmode algorithm to calculate the relationship between refractive index and temperature and established a photonic integrated circuit simulation model in the Photonic Integrated Circuit Simulator, importing relevant parameters. The circuit structure and simulation results are shown in Figure 8, Figure 9 and Figure 10. When no voltage is applied, the resonant wavelength of the MRR is 1543.29 nm. When a 2.677 V voltage is applied, the resonant wavelength of the MRR is tuned to 1547.80 nm.

#### 3.1.2. Nonlinear MRR

The nonlinear MRR proposed in Section 2.1 is achieved by incorporating a nonlinear factor into the waveguide. In the absence of incident light, its resonant state and behavior are identical to those of the linear MRR. When sufficient optical energy is applied, a carrier dispersion effect will occur within the waveguide, changing the waveguide refractive index and thus altering the resonant state of the MRR. In order to achieve two resonance states of 1543.29 nm and 1547.80 nm, the length of the nonlinear region is determined according to formula 1. The nonlinear coefficient of silicon (Si) is 10^−14^ cm^2^/W~10^−13^ cm^2^/W. When the input optical power is approximately 10 mW and the nonlinear length is 15.3545 um, the waveguide refractive index will undergo a change of −0.0507, leading to a π phase shift. Consequently, this induces a resonant peak shift of 4.51 nm, which corresponds to half of the Free Spectral Range (FSR).

(5) $$\left\{\begin{array}{l} \Delta \varphi =2\times \pi \times \Delta n\times L/\lambda \\ \Delta n=-(8.8\times e^{-22}\times M+8.5\times e^{-22}\times M^{0.8})\\ M=\gamma_{Si}\times \tau_{p}^{2}\times P_{avg}^{2}/(2\times hv_{pump}\times \sqrt{\pi }\times t\times S_{eff}^{2}) \end{array}\right.$$

Here, Δφ denotes the change in phase, Δn denotes the change in refractive index, M signifies the carrier concentration, $\gamma_{Si}$ represents the nonlinear coefficient of silicon, $\tau_{p}$ is the pulse duration of the pump light, $P_{avg}$ indicates the average power of the pump light, h is Planck’s constant, $v_{pump}$ is the frequency of the pump light, t refers to the thickness of the waveguide, and $S_{eff}$ denotes the effective mode area of the waveguide.

**Figure 11:**
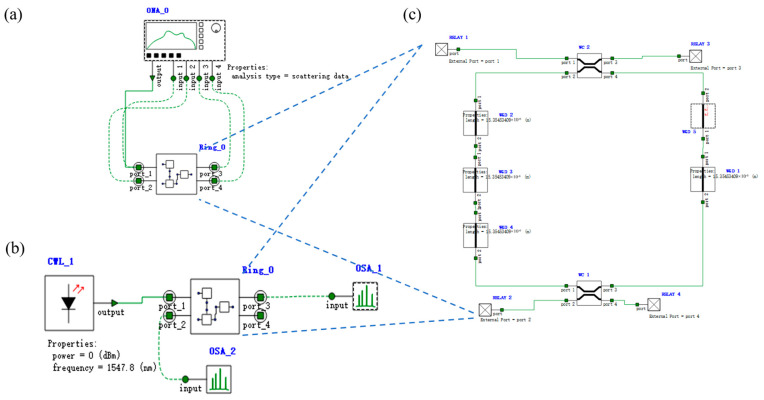
The model for nonlinear MRR. (**a**) Frequency-domain simulation. (**b**) Time-domain simulation. (**c**) Structure of nonlinear MRR. OSA: Optical spectral analyzer. CWL: Continuous wave laser. Ring: Micro-ring resonator. WGD: Straight waveguide. WC: Waveguide directional coupler.

**Figure 12:**
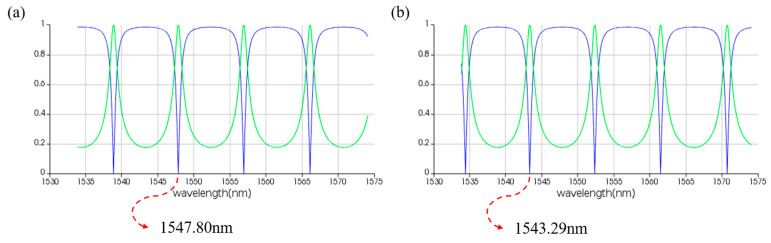
The spectral transmission results for nonlinear MRR in the presence of pumping. (**a**) Without thermal tuning. (**b**) With thermal tuning.

**Figure 13:**
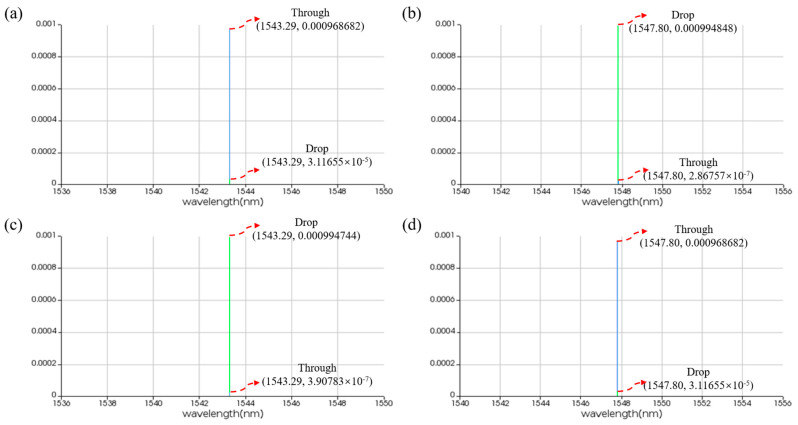
Time-domain simulation results for nonlinear MRR in the presence of pumping. (**a**,**b**) THROUGH and DROP port without thermal tuning. (**c**,**d**) THROUGH and DROP port with thermal tuning. The horizontal axis represents the wavelength unit in nm, and the vertical axis represents the output power unit in W.

The corresponding simulation structure is depicted in Figure 11, while the simulation results are shown in Figure 12 and Figure 13. In the initial state without thermal tuning, the resonant wavelength of the MRR is 1543.29 nm. When the incident light pumps the nonlinear MRR, the resonance peak shifts by 4.51 nm, resulting in a resonant wavelength of 1547.80 nm. Conversely, in the initial state with thermal tuning, the resonant wavelength of the MRR is 1547.80 nm. Upon excitation by incident light, the resonance peak again shifts by 4.51 nm, adjusting the resonant wavelength to 1543.29 nm. These simulation results demonstrate the feasibility of the nonlinear MRR design.

### 3.2. OSLU

#### 3.2.1. The OSLU of IS&NOT

**Figure 14:**
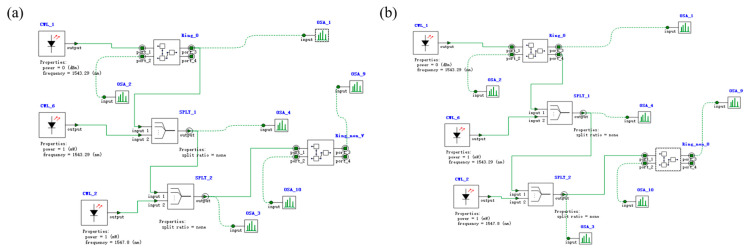
Model of IS&NOT-OSLU. (**a**) Structure of IS-OSLU with linear MRR without thermal tuning and nonlinear MRR with thermal tuning. (**b**) Structure of NOT-OSLU with linear MRR without thermal tuning and nonlinear MRR without thermal tuning. OSA: Optical spectral analyzer. CWL: Continuous wave laser. Ring: Micro-ring resonator. WGD: Straight waveguide. WC: Waveguide directional coupler. SPLT: Optical splitter/combiner.

Based on the IS&NOT-OSLU schematic diagram shown in Figure 2, the optical simulation structures are set up as illustrated in Figure 14. There is a continuous wave light source CWL_1 operating at wavelengths of 1543.29 nm or 1547.80 nm. As the input optical logic signal, it input to Ring_0_1, corresponding to the MRR A. The output acts on Ring_non_V, corresponding to the MRR B, while the input is provided by two other continuous wave optical sources, CWL_2 and CWL_3, whose operating wavelengths are 1543.29 nm and 1547.80 nm, respectively. The output signal is detected by optical spectrum analyzers OSA at the THROUGH port of Ring_non_V, used to characterize the logic function. The logic signal 0 is defined as light with a wavelength of 1543.29 nm, and the logic signal 1 is defined as light with a wavelength of 1547.80 nm.

**Figure 15:**
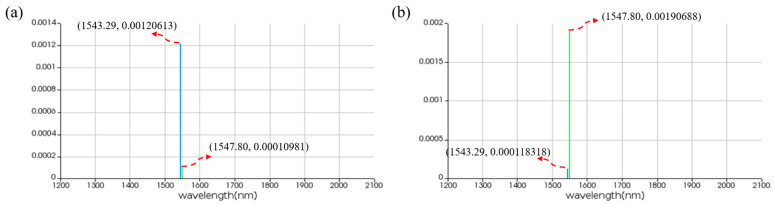
Simulation results of IS-OSLU at Through port. (**a**) When input wavelengths are 1543.29 nm. (**b**) When input wavelengths are 1547.80 nm. The horizontal axis represents the wavelength unit in nm, and the vertical axis represents the output power unit in W.

For the IS logic function, MRR A is a linear micro-ring resonator with a resonance wavelength of 1543.29 nm. MRR B is a nonlinear MRR with a resonance wavelength of 1543.29 nm in the presence of thermal tuning when light is acting upon it; otherwise, it shifts to 1547.80 nm. Simulation results are shown in Figure 15. When the logic signal is 0, i.e., the input light wavelength is 1543.29 nm, the Through port outputs light with a wavelength of 1543.29 nm, represented as logic signal 0. Conversely, when the logic signal is 1, i.e., the input light wavelength is 1547.80 nm, the Through port outputs light with a wavelength of 1547.80 nm, represented as logic signal 1. The simulation results indicate that the Through output port of OSLU under this kind of configuration successfully realizes the IS logic function.

**Figure 16:**
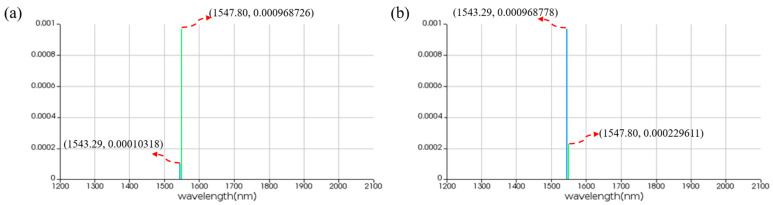
Simulation results of NOT-OSLU at Through port. (**a**) Input wavelengths are 1543.29 nm. (**b**) Input wavelengths are 1547.80 nm. The horizontal axis represents the wavelength unit in nm, and the vertical axis represents the output power unit in W.

For the NOT logic function, MRR A is a linear micro-ring resonator with a resonance wavelength of 1543.29 nm. MRR B is a nonlinear MRR with a resonance wavelength of 1547.80 nm in the absence of thermal tuning when light is acting upon it; otherwise, it shifts to 1543.29 nm. Simulation results are as shown in Figure 16. When the logic signal is 0, i.e., the input light wavelength is 1543.29 nm, the Through port outputs light with a wavelength of 1547.80 nm, represented as logic signal 1. Conversely, when the logic signal is 1, i.e., the input light wavelength is 1547.80 nm, the Through port outputs light with a wavelength of 1543.29 nm, represented as logic signal 0. The simulation results indicate that the Through output port of OSLU under this kind of configuration successfully realizes the NOT logic function.

#### 3.2.2. The OSLU of AND

**Figure 17:**
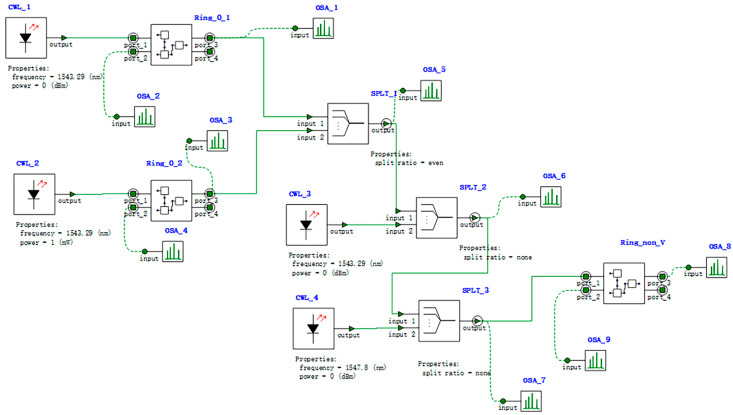
Model of AND-OSLU. Structure of AND-OSLU with linear MRR without thermal tuning and nonlinear MRR with thermal tuning. OSA: Optical spectral analyzer. CWL: Continuous wave laser. Ring: Micro-ring resonator. WGD: Straight waveguide. WC: Waveguide directional coupler. SPLT: Optical splitter/combiner.

Based on the AND-OSLU schematic diagram shown in Figure 3, the optical simulation structures are set up as illustrated in Figure 17. There are two continuous wave light sources CWL_1 and CWL_2 operating at wavelengths of 1543.29 nm or 1547.80 nm. As the input optical logic signals, they are simultaneously input to Ring_0_1 and Ring_0_2, corresponding to the MRR A1 and MRR A2. Their output acts on Ring_non_V, corresponding to the MRR B, while the input is provided by two other continuous wave optical sources, CWL_3 and CWL_4, whose operating wavelengths are 1543.29 nm and 1547.80 nm, respectively. The output signal is detected by optical spectrum analyzers OSA at the THROUGH port of Ring_non_V, used to characterize the logic function. The logic signal 0 is defined as light with a wavelength of 1543.29 nm, and the logic signal 1 is defined as light with a wavelength of 1547.80 nm.

**Figure 18:**
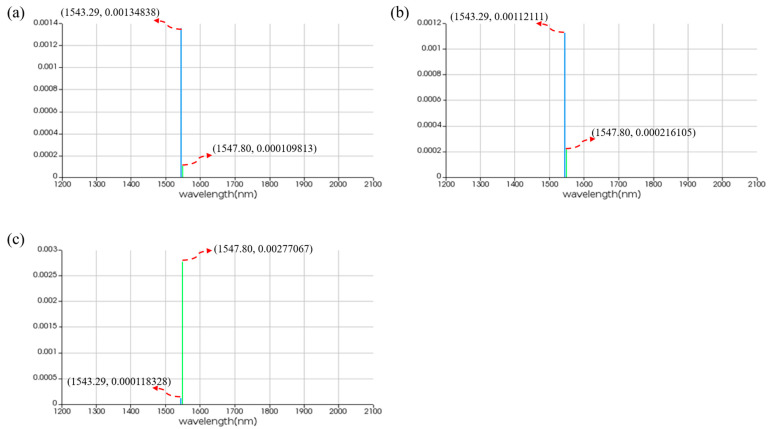
Simulation results of AND-OSLU at Through port. (**a**) Input wavelengths are 1543.29 nm and 1543.29 nm. (**b**) Input wavelengths are 1543.29 nm and 1547.80 nm. (**c**) Input wavelengths are 1547.80 nm and 1547.80 nm. The horizontal axis represents the wavelength unit in nm, and the vertical axis represents the output power unit in W.

For the AND logic function, MRR A1 and MRR A2 are linear micro-ring resonators with resonance wavelengths of 1543.29 nm. MRR B is a nonlinear MRR with a resonance wavelength of 1543.29 nm in the presence of thermal tuning when light is acting upon it; otherwise, it shifts to 1547.80 nm. Simulation results are shown in Figure 18. When the logic signal is 00, i.e., both input light wavelengths are 1543.29 nm, the Through port outputs light with a wavelength of 1543.29 nm, represented as logic signal 0. When the logic signal is 10 or 01, i.e., the input light wavelengths are 1543.29 nm and 1547.80 nm, the Through port outputs light with a wavelength of 1543.29 nm, represented as logic signal 0. When the logic signal is 11, i.e., both input light wavelengths are 1547.80 nm, the Through port outputs light with a wavelength of 1547.80 nm, represented as logic signal 1. The simulation results indicate that the Through output port of OSLU under this kind of configuration successfully realizes the AND logic function.

#### 3.2.3. The OSLU of OR

**Figure 19:**
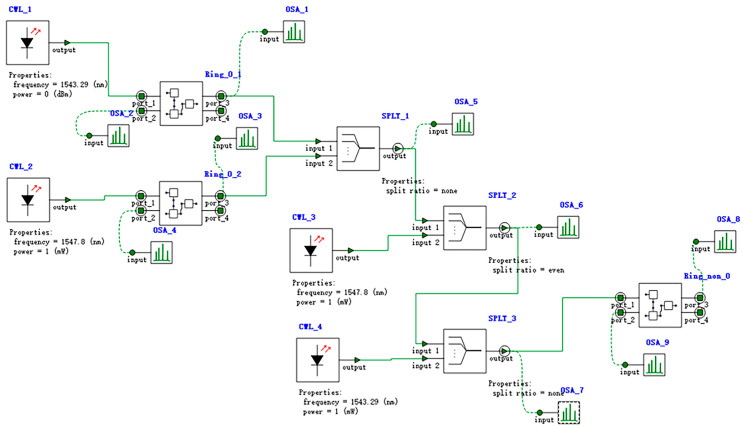
Model of OR-OSLU. Structure of OR-OSLU with linear MRR without thermal tuning and nonlinear MRR without thermal tuning. OSA: Optical spectral analyzer. CWL: Continuous wave laser. Ring: Micro-ring resonator. WGD: Straight waveguide. WC: Waveguide directional coupler. SPLT: Optical splitter/combiner.

Based on the OR-OSLU schematic diagram shown in Figure 4, the optical simulation structures are set up as illustrated in Figure 19. There are two continuous wave light sources CWL_1 and CWL_2 operating at wavelengths of 1543.29 nm or 1547.80 nm. As the input optical logic signals, they are simultaneously input to Ring_0_1 and Ring_0_2, corresponding to the MRR A1 and MRR A2. Their output acts on Ring_non_V, corresponding to the MRR B, while the input is provided by two other continuous wave optical sources CWL_3 and CWL_4, whose operating wavelengths are 1543.29 nm and 1547.80 nm, respectively. The output signal is detected by optical spectrum analyzers OSA at the DROP port of Ring_non_V, used to characterize the logic function. The logic signal 0 is defined as light with a wavelength of 1543.29 nm, and the logic signal 1 is defined as light with a wavelength of 1547.80 nm.

**Figure 20:**
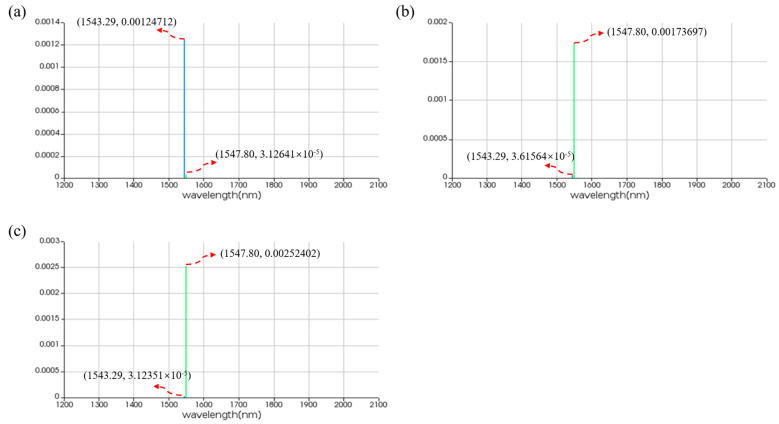
Simulation results of OR-OSLU at Through port. (**a**) Input wavelengths are 1543.29 nm and 1543.29 nm. (**b**) Input wavelengths are 1543.29 nm and 1547.80 nm. (**c**) Input wavelengths are 1547.80 nm and 1547.80 nm. The horizontal axis represents the wavelength unit in nm, and the vertical axis represents the output power unit in W..

For the OR logic function, MRR A1 and MRR A2 are linear micro-ring resonators with resonance wavelengths of 1543.29 nm. MRR B is a nonlinear MRR with a resonance wavelength of 1547.80 nm in the absence of thermal tuning when light is acting upon it; otherwise, it shifts to 1543.29 nm. Simulation results are shown in Figure 20. When the logic signal is 00, i.e., both input light wavelengths are 1543.29 nm, the Drop port outputs light with a wavelength of 1543.29 nm, represented as logic signal 0. When the logic signal is 10 or 01, i.e., the input light wavelengths are 1543.29 nm and 1547.80 nm, the Drop port outputs light with a wavelength of 1547.80 nm, represented as logic signal 1. When the logic signal is 11, i.e., both input light wavelengths are 1547.80 nm, the Drop port outputs light with a wavelength of 1547.80 nm, represented as logic signal 1. The simulation results indicate that the Through output port of OSLU under this kind of configuration successfully realizes the OR logic function.

### 3.3. Simulation Results of the Two-Input PPLA

**Figure 21:**
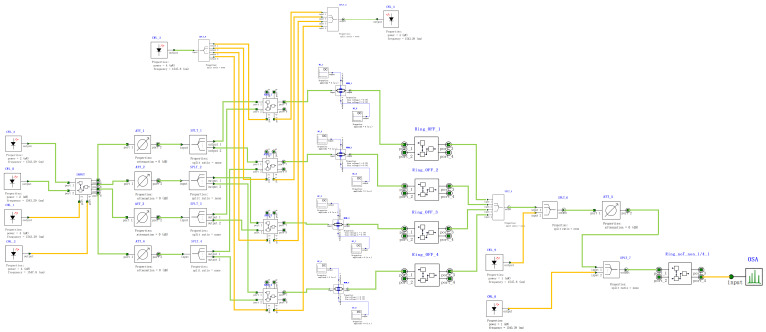
Optical circuit structure of two-input PPLA. OSA: Optical spectral analyzer. CWL: Continuous wave laser. Ring: Micro-ring resonator. WGD: Straight waveguide. WC: Waveguide directional coupler. SPLT: Optical splitter/combiner. ATT: Optical attenuator. MZM: Mach–Zehnder modulator. DC: DC source. AND: AND-OSLU composed of micro-ring resonators. INPUT: IS&NOT-OSLU composed of micro-ring resonators.

According to the PPLA schematic diagram shown in Figure 5, the corresponding optical simulation structures are set up as illustrated in Figure 21. Continuous wave light sources CWL_1 and CWL_2 firstly generate logical complementary input signals through IS&NOT-OSLU (INPUT unit, as depicted in the figure). Then, these input signals are fed into AND-OSLU (AND unit, also shown in the figure) to perform the AND logic computing, resulting in four minimum terms. Subsequently, the MZI-ARRAY (MZM units, illustrated in the figure) is driven by DC source. By leveraging its distinct switching states, the MZI-ARRAY provides varied inputs to the OR-OSLU. Ultimately, the OR-OSLU completes the OR operation among any minimal terms, thereby realizing the desired logical functions. The OR-OSLU is a four-input unit, which consists of four Ring_OFF elements corresponding to MRR A, as well as one Ring_noT_non_1/4_1 element corresponding to MRR B (as depicted in the figure). The drop port of the final MRR serves as the output for the entire optical logic structure, allowing for the observation of the optical power outputs at both wavelengths. In addition, an optical attenuator is also used to adjust the light intensity between each stage of the MRR, effectively balancing the input and output power at each stage. Similarly, the logic signal 0 is defined as light with a wavelength of 1543.29 nm and the logic signal 1 is defined as light with a wavelength of 1547.80 nm.

**Figure 22:**
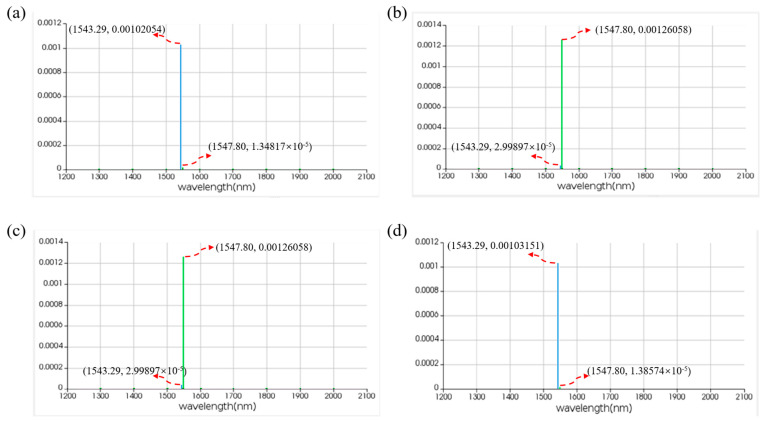
Simulation results of two-input PPLA for logical function of A⊕B. (**a**) Input wavelengths are 1543.29 nm and 1543.29 nm. (**b**) Input wavelengths are 1543.29 nm and 1547.80 nm. (**c**) Input wavelengths are 1547.80 nm and 1543.29 nm. (**d**) Input wavelengths are 1547.80 nm and 1547.80 nm. The horizontal axis represents the wavelength unit in nm, and the vertical axis represents the output power unit in W.

Figure 22 displays the simulation results for the logic function of A⊕B. When the logic signal is 00, i.e., both input light wavelengths are 1543.29 nm, the output port emits light at a wavelength of 1543.29 nm, resulting in an output logic signal of 0. When the logic signal is 01 or 10, i.e., the input light wavelengths are 1543.29 nm and 1547.80 nm, the output port emits light at the wavelength of 1547.80 nm, resulting in an output logic signal of 1. When the logic signal is 11, i.e., both input light wavelengths are 1547.80 nm, the output port emits light at the wavelength of 1543.29 nm, resulting in an output logic signal of 0. The simulation results indicate that the output port of PPLA successfully realizes the logic function of A⊕B.

**Figure 23:**
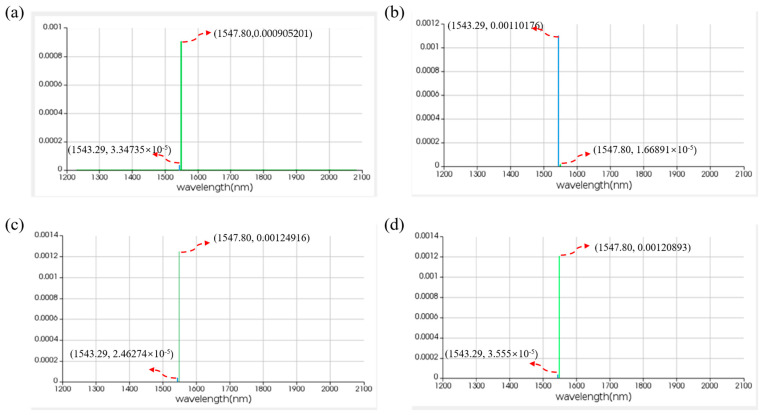
Simulation results of two-input PPLA for logical function of $A+\bar{B}$. (**a**) Input wavelengths are 1543.29 nm and 1543.29 nm. (**b**) Input wavelengths are 1543.29 nm and 1547.80 nm. (**c**) Input wavelengths are 1547.80 nm and 1543.29 nm. (**d**) Input wavelengths are 1547.80 nm and 1547.80 nm. The horizontal axis represents the wavelength unit in nm, and the vertical axis represents the output power unit in W.

Figure 23 demonstrates the simulation results for the logic function of $A+\bar{B}$. When the logic signal is 00, i.e., both input light wavelengths are 1543.29 nm, the output port emits light at a wavelength of 1547.80 nm, resulting in an output logic signal of 1. When the logic signal is 01, i.e., the input light wavelengths are 1543.29 nm and 1547.80 nm, the output port emits light at a wavelength of 1543.29 nm, resulting in an output logic signal of 0. When the logic signal is 10, i.e., the input light wavelengths are 1547.80 nm and 1543.29 nm, the output port emits light at a wavelength of 1547.80 nm, resulting in an output logic signal of 1. When the logic signal is 11, i.e., both input light wavelengths are 1547.80 nm, the output port emits light at a wavelength of 1547.80 nm, resulting in an output logic signal of 1. The simulation results indicate that the output port of PPLA successfully realizes the logic function of $A+\bar{B}$.

Table 1, Table 2 and Table 3 present the outcomes of all conceivable logic calculations, providing a detailed breakdown of the optical power outputs at the output ports along with their corresponding logic levels. In these tables, the VARIABLE section represents the logical combination of two input signals A and B, including 00, 01, 10, and 11. The RESULT section represents the output logic results, including the corresponding logic level, output wavelength, and output power level. Among them, m_1_, m_2_, m_3_, and m_4_ represent the four minimum terms, namely $\bar{A}\bar{B}$, $\bar{A}B$, $A\bar{B}$, AB. Table 1 shows the logical outputs constructed from two types of minimum terms, which can achieve logical functions $\bar{A}$, $\bar{B}$, A⊙B, A⊕B, B, A. When facing different input logic combinations, the probability of the corresponding output result being logic 1 or 0 is the same. When the output is logic 0, the power level of light with an output wavelength of 1543.29 nm ($\lambda_{1}$) is greater than 0.95 mW; when the output is logic 1, the power level of light with an output wavelength of 1547.80 nm ($\lambda_{2}$) is greater than 0.73 mW. Table 2 illustrates the logical outputs formed by the three types of minimum terms, which can achieve logical function $\bar{A}+\bar{B}$, $\bar{A}+B$, $A+\bar{B}$, A+B. For different input logic combinations, only one set of logic inputs produces an output of logic 0, while the other three sets of inputs produce an output of logic 1. When the output is logic 0, the power level of light with an output wavelength of 1543.29 nm ($\lambda_{1}$) is greater than 1 mW; when the output is logic 1, the power level of light with an output wavelength of 1547.80 nm ($\lambda_{2}$) is greater than 0.77 mW. Table 3 describes the logical output composed of four types of minimum terms. Since it is a combination of all minimum terms, the output is always logical 1, regardless of the type of logical input. The power level of light with an output wavelength of 1547.80 nm ($\lambda_{2}$) is greater than 0.8 mW. These findings robustly substantiate the validity and practicality of the logic array proposed in this paper, corroborating its theoretical integrity and its readiness for practical applications.

Note that the utilization of optical attenuators (OAs) and Mach–Zehnder Interferometers (MZIs) is also required in our design, which plays a critical role in the system link simulation. The optical attenuator primarily coordinates the input/output energy balance between circuits, while the MZI implements the programmable function, serving as an optical switch. In order to make the logic array function programmable, a scripting language approach is employed. This involved setting the power threshold and attenuation parameters of the attenuator based on the results obtained from the spectrum analyzer OSA, as well as setting the bias voltage of the MZI according to the required selection and combination.

## 4. Conclusions

In this study, we propose a programmable photonic logic array (PPLA) based on wavelength-type optical standard logic units (OSLUs). First, by designing a nonlinear micro-ring resonator (MRR) as a basic unit, the OSLU architecture is proposed to implement three basic optical logic calculations. Then, by cascading OSLUs, a two-input PPLA system is constructed to achieve any logic function. The simulation results from the Photonic Integrated Circuit Simulator demonstrate the effectiveness of OSLUs and PPLA. OSLUs achieve basic logic functions with a simple structure and good performance, and the PPLA, built with OSLUs as basic building blocks, is flexible, programmable, and can be reconfigured to perform different logic functions, showing a high degree of versatility and scalability. The OSLUs and PPLA proposed in this paper provide a highly flexible and reconfigurable optical platform, bringing new possibilities to the development of optical computing and photonic integrated circuits, and also providing strong support for the application of photonics in multiple fields.

In the future, we will explore the implementation and optimization of optical structures and systems with more complex, multioutput logic functions, search for simple and flexible reconfigurable schemes and self-configuring algorithms, make full use of multiple dimensions such as wavelength, polarization, and intensity to enhance the system capacity, and deepen the potential of the application of all-optical logic in optical communication and high-performance optical computing.

## Figures and Tables

**Table 1:** Logical combination results of two-input photonic logic array for two minimum terms.

VARIABLE		RESULT							
A	B		Function	m_1_ + m_2_	m_1_ + m_3_	m_1_ + m_4_	m_2_ + m_3_	m_2_ + m_4_	m_3_ + m_4_
		Characterization		$\bar{A}$	$\bar{B}$	A⊙B	A⊕B	B	A
0	0	Logic		1	1	1	0	0	0
		Wavelength/nm		1547.80	1547.80	1547.80	1543.29	1543.29	1543.29
		Power/mW		0.73873	0.73873	0.874803	1.02054	0.99038	0.99038
0	1	Logic		1	0	0	1	1	0
		Wavelength/nm		1547.80	1543.29	1543.29	1547.80	1547.80	1543.29
		Power/mW		0.761208	1.00962	1.02431	1.26058	0.743831	0.994258
1	0	Logic		0	1	0	1	0	1
		Wavelength/nm		1543.29	1547.80	1543.29	1547.80	1543.29	1547.80
		Power/mW		1.00917	1.03859	1.02561	1.26058	0.969297	0.978895
1	1	Logic		0	0	1	0	1	1
		Wavelength/nm		1543.29	1543.29	1547.80	1543.29	1547.80	1547.80
		Power/mW		1.0151	1.0151	1.46462	1.03151	1.13397	1.13397

**Table 2:** Logical combination results of two-input photonic logic array for three minimum terms.

VARIABLE		RESULT					
A	B		Function	m_1_ + m_2_ + m_3_	m_1_ + m_2_ + m_4_	m_1_ + m_3_ + m_4_	m_2_ + m_3_ + m_4_
		Characterization		$\bar{A}+\bar{B}$	$\bar{A}+B$	$A+\bar{B}$	A+B
0	0	Logic		1	1	1	0
		Wavelength/nm		1547.80	1547.80	1547.80	1543.29
		Power/mW		0.773708	0.905201	0.905201	1.08678
0	1	Logic		1	1	0	1
		Wavelength/nm		1547.80	1547.80	1543.29	1547.80
		Power/mW		0.936886	1.05891	1.10176	0.917892
1	0	Logic		1	0	1	1
		Wavelength/nm		1547.80	1543.29	1547.80	1547.80
		Power/mW		0.884492	1.04617	1.24916	1.26968
1	1	Logic		0	1	1	1
		Wavelength/nm		1543.29	1547.80	1547.80	1547.80
		Power/mW		1.1199	1.20893	1.20893	0.948801

**Table 3:** Logical combination results of two-input photonic logic array for four minimum terms.

VARIABLE		RESULT		
A	B		Function	m_1_ + m_2_ + m_3_ + m_4_
		Characterization		1
0	0	Logic		1
		Wavelength/nm		1547.80
		Power/mW		0.949655
0	1	Logic		1
		Wavelength/nm		1547.80
		Power/mW		0.973804
1	0	Logic		1
		Wavelength/nm		1547.80
		Power/mW		1.36479
1	1	Logic		1
		Wavelength/nm		1547.80
		Power/mW		0.800419

## Data Availability

Data underlying the results presented in this paper are not publicly available at this time but may be obtained from the authors upon reasonable request.
